# Supplementary material for: Human embryonic stem cell-derived cardiomyocyte platform screens inhibitors of SARS-CoV-2 infection
Source: Commun Biol. 2021 Jul 29;4:926. doi: 10.1038/s42003-021-02453-y (PMC8322398; doi:10.1038/s42003-021-02453-y)
Supplement: Supplementary file 1 — Supplementary Information [file 42003_2021_2453_MOESM1_ESM.pdf]

Supplementary Information

Human embryonic stem cell-derived cardiomyocyte platform screens inhibitors of SARS-CoV-2 infection

Thomas L. Williams[1§], Maria T. Colzani[2§], Robyn G.C. Macrae[1,2], Emma L. Robinson[3], Stuart Bloor[4], Edward J. D. Greenwood[4], Jun Ru Zhan[4], Gregory Strachan[5], Rhoda E. Kuc[1], Duuamene Nyimanu[1], Janet J. Maguire[1], Paul J. Lehner[4], Sanjay Sinha[2#], Anthony P. Davenport[1#*]

1. Experimental Medicine and Immunotherapeutics, University of Cambridge, Addenbrooke's Hospital, Cambridge, U.K.
2. Wellcome-MRC Cambridge Stem Cell Institute, Jeffrey Cheah Biomedical Centre, University of Cambridge, Cambridge, U.K.
3. School of Medicine, Division of Cardiology, University of Colorado Denver, Anschutz Medical Campus, Aurora, U.S.A.
4. Cambridge Institute of Therapeutic Immunology & Infectious Disease (CITIID), Jeffrey Cheah Biomedical Centre, University of Cambridge, Cambridge, U.K.
5. Wellcome Trust-MRC Institute of Metabolic Science, Metabolic Research Laboratories, Addenbrooke's Biomedical Campus, Cambridge, U.K.

[§]Joint first authors, contributed equally. [#]Joint senior authors.

Author for Correspondence:
*Anthony P. Davenport,
Experimental Medicine and Immunotherapy,
University of Cambridge,
Level 6, Addenbrooke's Centre for Clinical Investigation,
Box 110, Addenbrooke's Hospital,
Cambridge, CB2 0QQ, U.K.
Tel:+44(0)1223 336899
apd10@medschl.cam.ac.uk

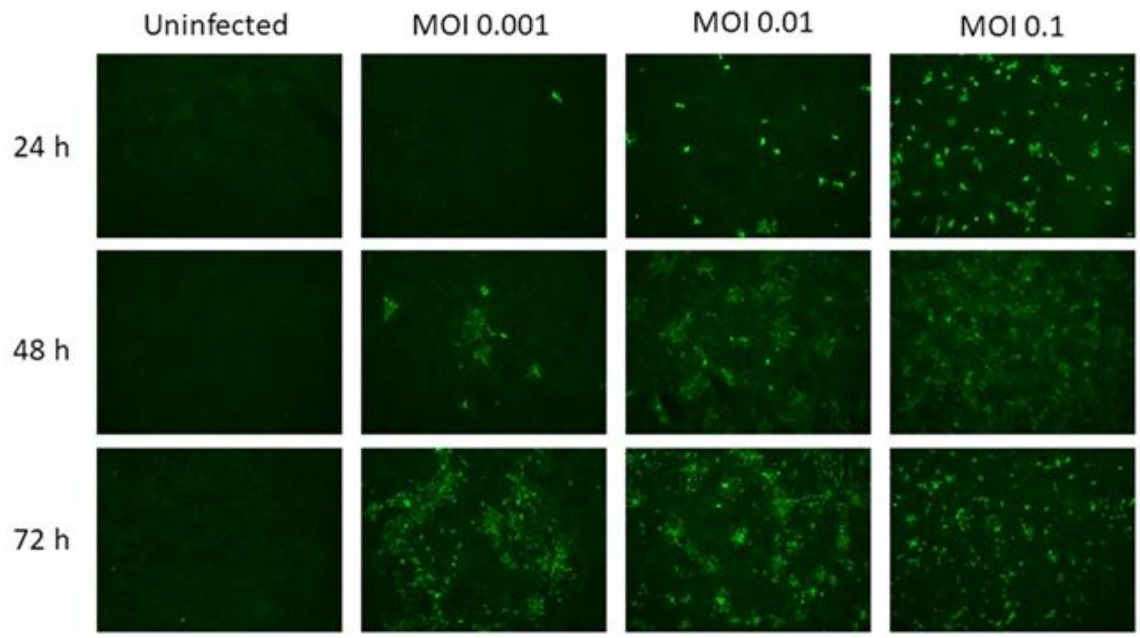

Supplementary Fig. 1: Titre- and time-dependent SARS-CoV-2 infection of hESC-derived cardiomyocytes. Representative fluorescent images (n=2 independent experiments) of human embryonic stem cell-derived cardiomyocytes (hESC-CMs) infected with SARS-CoV-2 at several different viral titres ((0.001-0.1 multiplicity of infection (MOI)), or left untreated, and incubated for different time periods (24-72 h). Infected cells were immunolabelled after fixation with 2% formaldehyde and permeabilization with BD Perm/Wash buffer (BD Biosciences, 554723) using sheep anti-SARS-CoV-2 nucleocapsid antibody (DA114, MRC-PPU). Cells were visualised using a donkey anti-sheep secondary antibody conjugated to Alexa Fluor 488 (green) (Jackson ImmunoResearch #713-545-147).

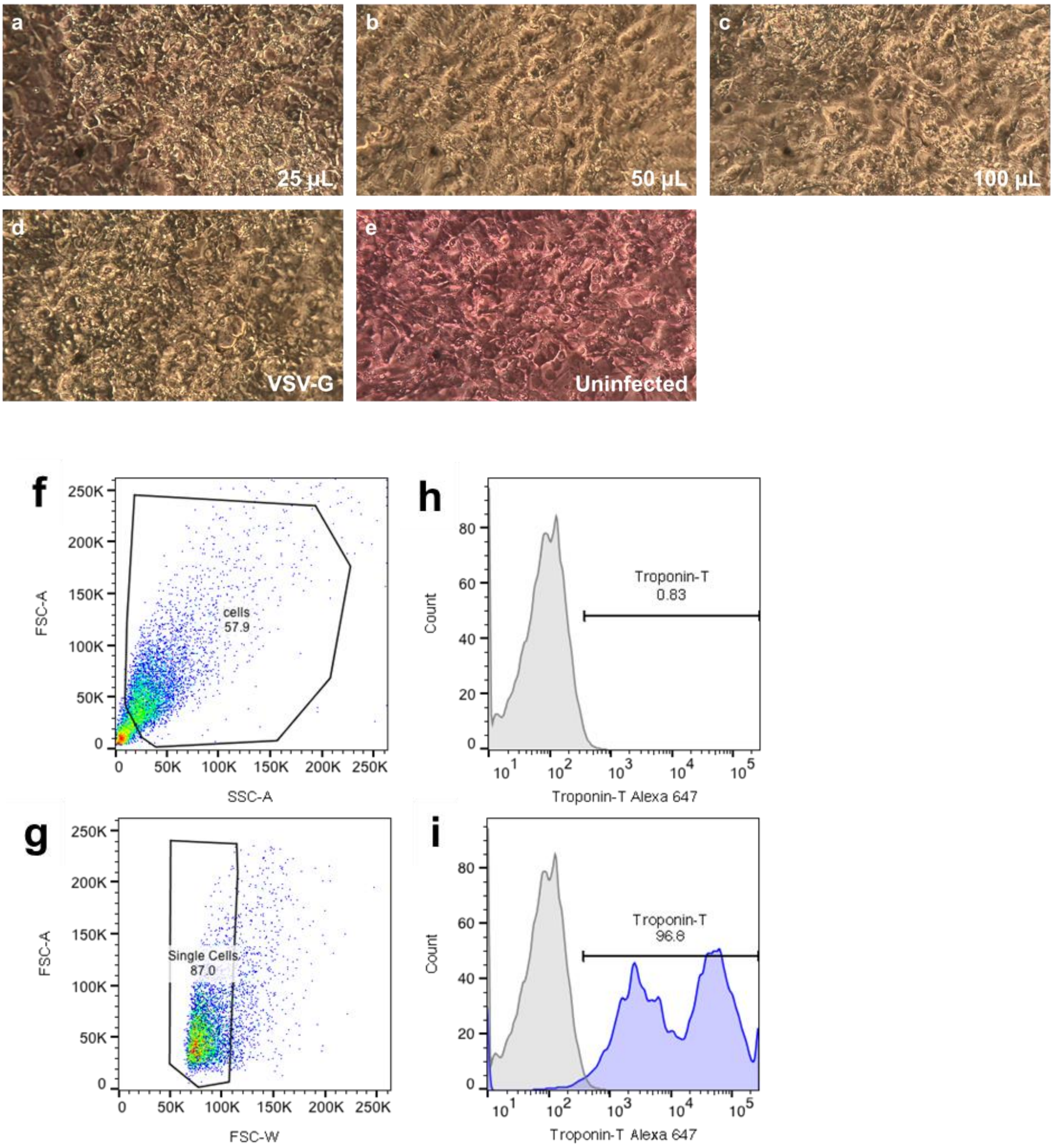

Supplementary Fig. 2: Cell populations of pure, beating cardiomyocytes. a-e Representative brightfield video files of beating human embryonic stem cell-derived cardiomyocytes (hESC-CMs) following infection with 25 µL (**a**), 50 µL (**b**), or 100 µL (**c**) SARS-CoV-2 spike pseudotyped lentivirus. Control cells were treated with 100 µL vesicular stomatitis virus (VSV-G) pseudotyped lentivirus (**d**) or left untreated with viral particles (**e**). **f-i** hESC purity flow cytometry data. **f** Cells were gated using FSC and SSC. **g** Single cells were distinguished from doublets and aggregates by plotting FSC-A against FSC-W. Subsequent histograms were gated on the single cells population. **h** The Troponin-T gate was determined using cells stained with IgG control. **i** Representative plot displaying purity of hESC-CM differentiation by flow cytometry using an antibody specific for cardiac troponin-T (96.8 % of the population shown to be positive for this marker).

Compound:	Target:	Type:	Concentration used:
Camostat	TMPRSS2	Small molecule inhibitor	30 µM
Benztropine	B^0AT1	Small molecule inhibitor	30 µM
E64d	Cathepsin B/L	Small molecule inhibitor	30 µM
DX600	ACE2	Peptide inhibitor	10 µM
ACE2 ab	ACE2	Polyclonal antibody (neutralizing)	20 µg/mL

Supplementary Table. 1: Compounds used, their protein targets, and other relevant information. The table shows the compounds used in screening experiments at the denoted concentrations, and their respective protein targets shown to be expressed in the human embryonic stem cell-derived cardiomyocytes (hESC-CMs) cell model, as well as adult cardiomyocytes. Concentrations used in the hESC-CM screen were chosen based on the pIC50 values reported in literature or on IUPHAR/BPS Guide to PHARMACOLOGY (https://www.guidetopharmacology.org).
