# Supplementary material for: Human embryonic stem cell-derived cardiomyocyte platform screens inhibitors of SARS-CoV-2 infection
Source: Commun Biol. 2021 Jul 29;4:926. doi: 10.1038/s42003-021-02453-y (PMC8322398; doi:10.1038/s42003-021-02453-y)
Supplement: Supplementary file 2 — Description of Additional Supplementary Files [file 42003_2021_2453_MOESM2_ESM.pdf]

Description of Additional Supplementary Files

File name: Supplementary data 1

Description: Source data.

File name: Supplementary video 1

Description: Representative brightfield video files of beating human embryonic stem cell-derived cardiomyocytes (hESC-CMs) following infection with 25 µL of SARS-CoV-2 spike pseudotyped lentivirus.

File name: Supplementary video 2

Description: Representative brightfield video files of beating human embryonic stem cell-derived cardiomyocytes (hESC-CMs) following infection with 50 µL of SARS-CoV-2 spike pseudotyped lentivirus.

File name: Supplementary video 3

Description: Representative brightfield video files of beating human embryonic stem cell-derived cardiomyocytes (hESC-CMs) following infection with 100 µL of SARS-CoV-2 spike pseudotyped lentivirus.

File name: Supplementary video 4

Description: Representative brightfield video files of beating human embryonic stem cell-derived cardiomyocytes (hESC-CMs). Control cells were treated with 100 µL vesicular stomatitis virus (VSV-G) pseudotyped lentivirus.

File name: Supplementary video 5

Description: Representative brightfield video files of beating human embryonic stem cell-derived cardiomyocytes (hESC-CMs). Control cells left untreated with viral particles.
