# Supplementary material for: Human embryonic stem cell-derived cardiomyocyte platform screens inhibitors of SARS-CoV-2 infection
Source: Commun Biol. 2021 Jul 29;4:926. doi: 10.1038/s42003-021-02453-y (PMC8322398; doi:10.1038/s42003-021-02453-y)
Supplement: Supplementary file 9 — Reporting Summary [file 42003_2021_2453_MOESM9_ESM.pdf]

nature research

Corresponding author(s): Professor Anthony P. Davenport

Last updated by author(s): 1/3/2021

Reporting Summary

Nature Research wishes to improve the reproducibility of the work that we publish. This form provides structure for consistency and transparency in reporting. For further information on Nature Research policies, see our Editorial Policies and the Editorial Policy Checklist.

Statistics

For all statistical analyses, confirm that the following items are present in the figure legend, table legend, main text, or Methods section.

n/a	Confirmed	
☐	☒	The exact sample size (*n*) for each experimental group/condition, given as a discrete number and unit of measurement
☐	☒	A statement on whether measurements were taken from distinct samples or whether the same sample was measured repeatedly
☐	☒	The statistical test(s) used AND whether they are one- or two-sided<br>*Only common tests should be described solely by name; describe more complex techniques in the Methods section.*
☒	☐	A description of all covariates tested
☐	☒	A description of any assumptions or corrections, such as tests of normality and adjustment for multiple comparisons
☐	☒	A full description of the statistical parameters including central tendency (e.g. means) or other basic estimates (e.g. regression coefficient) AND variation (e.g. standard deviation) or associated estimates of uncertainty (e.g. confidence intervals)
☒	☐	For null hypothesis testing, the test statistic (e.g. *F*, *t*, *r*) with confidence intervals, effect sizes, degrees of freedom and *P* value noted<br>*Give P values as exact values whenever suitable.*
☒	☐	For Bayesian analysis, information on the choice of priors and Markov chain Monte Carlo settings
☒	☐	For hierarchical and complex designs, identification of the appropriate level for tests and full reporting of outcomes
☒	☐	Estimates of effect sizes (e.g. Cohen's *d*, Pearson's *r*), indicating how they were calculated

Our web collection on statistics for biologists contains articles on many of the points above.

Software and code

Policy information about availability of computer code

Data collection	No software was used in collection of these data.
Data analysis	Statistical analysis was performed using commercially available Graphpad Prism and Harmony Analysis

For manuscripts utilizing custom algorithms or software that are central to the research but not yet described in published literature, software must be made available to editors and reviewers. We strongly encourage code deposition in a community repository (e.g. GitHub). See the Nature Research guidelines for submitting code & software for further information.

Data

Policy information about availability of data

All manuscripts must include a data availability statement. This statement should provide the following information, where applicable:

- Accession codes, unique identifiers, or web links for publicly available datasets
- A list of figures that have associated raw data
- A description of any restrictions on data availability

The data that support the findings of this study are available from the corresponding authors upon reasonable request.

Field-specific reporting

Life sciences study design

All studies must disclose on these points even when the disclosure is negative.

Sample size	Sample size has not been formally predetermined but was based on previous studies
Data exclusions	No data were excluded
Replication	Replication as indicated in manuscript for each experiment
Randomization	Not applicable
Blinding	Not applicable

Reporting for specific materials, systems and methods

We require information from authors about some types of materials, experimental systems and methods used in many studies. Here, indicate whether each material, system or method listed is relevant to your study. If you are not sure if a list item applies to your research, read the appropriate section before selecting a response.

Materials & experimental systems

n/a	Involved in the study	
☐	☒	Antibodies
☐	☒	Eukaryotic cell lines
☒	☐	Palaeontology and archaeology
☒	☐	Animals and other organisms
☒	☐	Human research participants
☒	☐	Clinical data
☒	☐	Dual use research of concern

Methods

n/a	Involved in the study	
☒	☐	ChIP-seq
☐	☒	Flow cytometry
☒	☐	MRI-based neuroimaging

Antibodies

Antibodies used	Primary goat polyclonal antibody to Human ACE-2 (AF933; R&D Systems; 1:100); primary rabbit monoclonal antibody to TMPRSS2 (ab92323; Abcam; 1:500); primary rabbit monoclonal antibody to B0AT1 (ab180516; Abcam; 1:300); primary rabbit monoclonal antibody to cathepsin B (ab125067; Abcam; 1:100); primary rabbit polyclonal antibody to cathepsin L (ab203028; Abcam; 1:100); or primary rabbit polyclonal antibody to furin (ab3467; Abcam; 1:500).<br>Secondary polyclonal Donkey Anti-Goat IgG H&L antibody conjugated to Alexa Fluor 555 (ab150130; Abcam; 1:200) or Donkey Anti-Rabbit IgG H&L antibody conjugated to Alexa Fluor 555 (ab150066; Abcam; 1:200).
Validation	Antibody validation provided by manufacturers is available from Abcam (https://www.abcam.com/) and R&D Systems (https://www.rndsystems.com/). Each antibody was tested using positive controls using 4% PFA fixed cryostat sections of heart sections. Images for control sections are displayed in the figures.

Eukaryotic cell lines

Policy information about cell lines

Cell line source(s)	The hESCs are H9s from Wicell, Madison. Full informed consent was obtained from Wicell who then distributed the cells. We have registered our use of these hESCs with the UK Stem Cell Bank - no further ethical approval is required to use the cells for in vitro studies.
Authentication	Cell lines were obtained directly from the provider. No formal form of authentication was carried out.
Mycoplasma contamination	Mycoplasma screening was performed on the cell lines and was negative.
Commonly misidentified lines (See ICLAC register)	Cell line used in this study is not listed in the ICLAC database.

Flow Cytometry

Plots

Confirm that:

- [x] The axis labels state the marker and fluorochrome used (e.g. CD4-FITC).
- [x] The axis scales are clearly visible. Include numbers along axes only for bottom left plot of group (a 'group' is an analysis of identical markers).
- [x] All plots are contour plots with outliers or pseudocolor plots.
- [x] A numerical value for number of cells or percentage (with statistics) is provided.

Methodology

Sample preparation	hESC-derived cardiomyocytes were collected as pellets and resuspended in Fixation/Solubilization solution (BD Cytofix/ Cytoperm Fixation/Permeabilization Kit, Biosciences) for 20 mins at 4°C. Cells were then pelleted by centrifugation, washed three times in phosphate buffered saline containing 0.1% BSA and 2 mM EDTA (PBE), then resuspended in PBE containing FITC-conjugated antibody specific for cardiac troponin T (Miltenyi Biotec, cat no. 130-119-575) at a concentration of 1:50 and incubated for 2 h at 4°C. Three washes were performed using PBE and then cells were resuspended in PBE and run on a Flow Cytometer.
Instrument	LSRFortessa X-20 Flow Cytometer (BD Biosciences)
Software	Flow cytometric analysis was performed with FlowJo VX software (BD Biosciences).
Cell population abundance	Purity of hESC-CM differentiation by flow cytometry using an antibody specific for cardiac troponin-T (96.8 % of the population shown to be positive for this marker). Consistent results were seen across differentiations.
Gating strategy	HESC-derived cardiomyocytes were gated on FSC/SSC first and an unstained sample, IgG isotype and secondary only as controls to establish the gate for positive cells.

- [x] Tick this box to confirm that a figure exemplifying the gating strategy is provided in the Supplementary Information.
